# Frequency of exposure to arboviruses and characterization of Guillain Barré syndrome in a clinical cohort of patients treated at a tertiary referral center in Brasília, Federal District

**DOI:** 10.1590/0037-8682-0306-2021

**Published:** 2022-04-08

**Authors:** Luíza Morais de Matos, Ariely Teotonio Borges, Aline Barbosa Palmeira, Vinicius Moreira Lima, Ernane Pires Maciel, Rubens Nelson Morato Fernandez, João Pedro Lima Mendes, Gustavo Adolfo Sierra Romero

**Affiliations:** 1 Universidade de Brasília, Faculdade de Medicina, Núcleo de Medicina Tropical, Brasília, DF, Brasil.; 2 Instituto Hospital de Base do Distrito Federal, Unidade de Infectologia, Brasília, DF, Brasil.; 3 Instituto Hospital de Base do Distrito Federal, Unidade de Neurologia, Brasília, DF, Brasil.

**Keywords:** Guillain Barré Syndrome, Arbovirus, Dengue, Clinical cohort, Diagnosis, Prognosis

## Abstract

**Background:**

Guillian Barré syndrome (GBS) is an acute autoimmune polyradiculoneuropathy often associated with previous exposure to infectious agents.

**Methods:**

A clinical cohort of 41 patients with GBS admitted to the Base Hospital Institute of the Federal District between May 2017 and April 2019 was followed up for 1 year. Serological tests for arbovirus detection and amplification of nucleic acids using polymerase chain reaction for zika virus (ZIKV), dengue virus (DENV), and chikungunya virus (CHIKV) were performed.

**Results:**

The cohort consisted of 61% men with a median age of 40 years, and 83% had GBS-triggering events. A total of 54% had Grade 4 disability, 17% had Grade 3, 12% had Grade 2, 10% had Grade 5, and 7% had Grade 1. The classic form occurred in 83% of patients. Nerve conduction evaluations revealed acute demyelinating inflammatory polyneuropathy (51%), acute motor axonal neuropathy (17%), acute sensory-motor neuropathy (15%), and indeterminate forms (17%). Four patients were seropositive for DENV. There was no laboratory detection of ZIKV or CHIKV infection. Ninety percent of patients received human immunoglobulin. Intensive care unit admission occurred in 17.1% of the patients, and mechanical ventilation was used in 14.6%. One patient died of Bickerstaff’s encephalitis. Most patients showed an improvement in disability at 10 weeks of follow-up.

**Conclusions:**

GBS in the Federal District showed a variable clinical spectrum, and it was possible to detect recent exposure to DENV.

## INTRODUCTION

Guillain Barré syndrome (GBS) comprises a group of heterogeneous disorders with acute onset and is one of the most common causes of acute flaccid paralysis worldwide. The main characteristic is bilateral muscle weakness associated with somatosensory changes, dysautonomia, hyporeflexia, and pain, reaching a nadir of severity in up to 4 weeks^1^. GBS occurs via activation of autoimmunity against the peripheral nervous system after various stimuli, often of infectious origin^2^ ^,^ ^3^. Affected patients generally have a good prognosis and recover within weeks after the onset of symptoms. However, approximately 5% of patients die from complications, including respiratory failure, pneumonia, and arrhythmias^4^ ^,^ ^5^ ^,^ ^6^. 

GBS diagnosis is based on a combination of characteristics known as the Brighton criteria^7^. There is a cerebrospinal fluid (CSF) pattern of the disease, which may be normal at the onset but exhibits an increase in total proteins with a normal nucleated cell count characterized by protein- or albumin-cytological dissociation^8^.

GBS is classified into clinical variants, including classical GBS, pharyngo-cervico-brachial (FCB), paraparetic, facial diparesis form, Miller-Fisher syndrome (SMF), Bickerstaff encephalitis (BE), and overlaps between the variants^9^.

Nerve conduction (NEC) studies have revealed the following subtypes: acute demyelinating inflammatory polyneuropathy (AIDP), acute motor axonal neuropathy (AMAN), acute sensory-motor neuropathy (AMSAN), and indeterminate pattern^10^ ^.^

Infectious and noninfectious environmental agents in genetically susceptible hosts trigger disease development. Zika virus (ZIKV) infection, a ribonucleic acid (RNA) flavivirus transmitted by mosquitoes of the genus *Aedes*, was identified as a potential trigger for GBS^5^. ZIKV causes a self-limited disease that may be asymptomatic or present with skin rash, gastrointestinal disorders, fever, arthralgia, headache, conjunctivitis^11^, and occasionally congenital microcephaly, para-infectious, and post-infectious GBS^12^. An increased incidence of GBS was reported concomitantly with the ZIKV epidemic, and a relationship between arboviruses and GBS has been observed^6^. Encephalitis and GBS were also related to dengue virus (DENV) and chikungunya virus (CHIKV) progression, with long-term sequelae and expressive abnormalities in radiological examinations in patients with brain disorders^13^ ^,^ ^14^ ^,^ ^15^.

Treatment includes intravenous human immunoglobulin (IVIg) and plasmapheresis, which accelerate recovery^16^.

Despite the increased rates of GBS associated with the ZIKV outbreak^17^ ^,^ ^18^, no studies have examined the clinical characterization of GBS and its long-term evolution in the Federal District, and no systematic investigation of exposure to arboviruses DENV, ZIKV, and CHIKV have been performed. Therefore, the present study aimed to describe a clinical cohort of patients with GBS treated at a tertiary-level referral center and explore the exposure to three arboviruses that circulate in a sympatric manner in the Federal District.

## METHODS

The study was nested in the *Zika and other Arbovirus Infections Cohort Studies* initiative of the Center of Tropical Medicine of the University of Brasilia, within the framework of the research project *Natural History of ZIKV Infection in the Federal District.* This project was designed to analyze the clinical, epidemiological, and immunological data on ZIKV infection and other arboviruses in the general population, pregnant women, and live births in the Federal District in a scenario of sympatric circulation of DENV and CHIKV viruses and high vaccination coverage against yellow fever.

The sample was a clinical cohort of patients admitted with suspected GBS at the Instituto Hospital de Base do Distrito Federal (IHBDF), a tertiary-level referral unit and the largest public hospital in the Federal District, from May 2017 to May 2019. Because GBS is a relatively rare clinical entity, the sample was defined for the universal patients who consulted for suspected clinical conditions of GBS and were referred to the IHBDF neurological emergency unit from May 2017 to April 2019.

The level of diagnostic certainty was based on clinical and laboratory data classified from 1 to 3 according to the case definitions of the *Brighton Collaboration* in the context of the Zika virus Interim Guidance (WHO)^7^. The following inclusion criteria were used for the cases: GBS diagnosis according to the Brighton diagnostic criteria, onset of symptoms within 4 weeks preceding the consultation and signing the informed consent form (ICF). In addition, cases with a confirmed etiology other than GBS were excluded. The clinical variants of GBS were Miller Fisher syndrome, pharyngeal-cervical-brachial weakness, paraparetic GBS, bifacial weakness with paresthesia, Bickerstaff’s brainstem encephalitis with subtypes, and possible overlaps (adapted Wakerley classification)^9^.

The collected data included epidemiological characteristics, prodromal symptoms, diagnostic tests for GBS, clinical characteristics and signs of severity, treatment, and time to symptom improvement. The evaluation consisted of interviews, physical examination, and neurological evaluation using the standardized GBS Outcome Study-ZIKA (IGOS)^19^ ^,^ ^20^. Aspects of the acute phase and progression of GBS, and the presence of signs and symptoms related to arboviruses, were evaluated in an interview. The physical examination included data on stance, gait assessment, muscle bulk, tone, limb strength and reflexes, coordination, posture, changes in sensory function, involvement of the cranial nerves, involvement of the autonomic system, severity indicators, intensive care unit (ICU) admission, mechanical ventilation, and complications. Previous potential triggering GBS events that occurred 4 weeks before neurological onsets, such as diarrhea, flu, vaccination, and symptoms related to infections by DENV, ZIKV, and CHIKV, such as exanthema, arthralgia, fever, and diarrhea, were also recorded. A neurologist performed clinical examinations upon admission and 1-2, 4-8, 13, 26, and 52 weeks after admission.

The patients were subjected to the following examinations during hospitalization: CSF analysis (cytology, protein); serological tests for arbovirus detection; and amplification of nucleic acids using polymerase chain reaction (PCR) for ZIKV, DENV, and CHIKV in the CSF, serum, and urine samples. Briefly, the serological test was immunoglobulin M antibody capture enzyme-linked immunosorbent assay (MAC-ELISA), and the molecular test was real-time quantitative PCR (RT-qPCR) using the TaqMan® system with probes and primers with previously defined oligonucleotide sequences from a published Centers for Disease Control and Prevention (CDC) protocol^21^ ^22^.

A nerve conduction study (NEC) included the following subtypes: acute demyelinating inflammatory polyneuropathy (AIPD), AMAN, AMSAN, and indeterminate pattern based on the Asbury and Cornblath criteria^23^. During follow-up, clinical or laboratory data were obtained in addition to the electronic medical records used in the IHBDF and during the recent coronavirus disease 2019 (COVID-19) panic via a video conference.

The follow-up flow was as follows: admission and emergency care of the IHBDF by the neurology team on duty; performing the clinical diagnosis of GBS and requesting lumbar puncture to collect CSF for laboratory diagnosis of GBS; offer and signature of the TCLE; a collection of venous blood and urine samples; ordering tests of biological samples to the Central Laboratory of Public Health of the Federal District (LACEN DF); neurological evaluation using the standardized form GBS Outcome Study-ZIKA (IGOS) upon admission and at 1-2, 4-8, 13, 26, and 52 weeks; and performance of electroneuromyography during hospitalization in the neurology ward.

Data were recorded on the Redcap online platform and made available in Microsoft Excel spreadsheets. Categorical variables were analyzed as raw frequencies and proportions, and normally distributed continuous variables were expressed as means or medians, with the corresponding measures of dispersion. The frequency of arbovirus infection in the studied samples was expressed as a percentage. Survival analysis was performed using the *Statistical Package for Social Sciences* (SPSS) v. 21. Estimates were obtained using the Kaplan-Meier method. Survival rates were compared between groups using the log-rank test with a 5% significance level for decision making. The outcome of interest in the survival analysis was the improvement defined by the reducing at least one point in the classification of the degree of disability over the 52-week follow-up period. Participants who did not exhibit the outcome of interest until week 52 were censored and the time of the last evaluation performed during the follow-up period was used as a reference. The estimates of the time to outcome are expressed as medians with their respective 95% confidence intervals (CI). All analyses were performed using the International Business Machine (IBM) SPSS, version 21. The study followed the recommendations for research involving humans, including the Council National Health Board Resolution No. 466 of December 12, 2012 (DOU, 2013, Section 1. n°12) and the Declaration of Helsinki^24^. All the participants provided written informed consent. The research ethics committee of the Faculty of Medicine of the University of Brasília (CAAE 1.989.868) and the Foundation for Teaching and Research in Health Sciences/State Health Secretariat, Federal District (CAAE 1.910.150) approved this study.

## RESULTS

Forty-eight patients were considered candidates as participants in the study. Seven candidates were excluded: five due to alternative diagnoses, one due to death before signing the ICF, and one who did not meet the Brighton criteria. Therefore, only 41 patients were included in this study. Table 1 describes the clinical and laboratory characteristics and assessment of nerve conduction upon admission, treatment provided, and observed complications during hospitalization.

**TABLE 1:** Characteristics of 41 patients with GBS at admission treated at a tertiary referral center from May 2017 to April 2019 in the Federal District, Brazil.

**Characteristic**	Frequency	%
Median degree of disability at admission *	4	
Grade 1	3	7.3
Grade 2	5	12.2
Grade 3	7	17.1
Grade 4	22	53.6
Grade 5	4	9.8
Involvement of cranial nerves	22	53.6
Oculomotor nerves	3	7.3
Facial nerves	14	34.1
Bulbar nerves	12	29.3
Other **	3	7.3
Autonomic dysfunction	15	36.6
Cardiac (arrhythmia, sustained tachycardia or bradycardia, and cardiac arrest)	2	4.9
Blood pressure (fluctuations, hypertension, and hypotension)	8	19.5
Gastroenteric	3	7.3
Bladder disfunction	7	17.1
Sensory deficit	22	53.6
Pain	19	46.3
Clinical variants		
Classical	34	82.9
Form pharyngo-cervical-brachial	1	2.4
Miller Fisher syndrome	1	2.4
Miller Fisher-SGB overlap syndrome	3	7.3
SMF + pharyngo-cervical-brachial overlap syndrome	1	2.4
Bickerstaff’s encephalitis	1	2.4
Previous triggering events	34	82.9
Infection of the upper respiratory tract	16	39.0
Gastroenteritis	13	31.7
Vaccination	4	9.8
Other ***	7	17.1
Days between triggering event and onset of weakness		
0-7	17	41.5
8-14	7	17.1
15-21	2	4.9
22-28	1	2.4
29-35	1	2.4
Previous episode of GBS	2	4.9
Examination of the CSF	40	97.6
Cellularity <5	38	92.7
Cellularity 5-50/µL	2	4.9
Cellularity >50/µL	0	0
Protein concentration >0.45 g/L	18	43.9
Median number of days between onset of symptoms and CSF examination	6	
Electrophysiological classification		
AMAN	9	23
AMSAN	8	20.5
AIDP	21	53.8
Indeterminate	1	2.5
Specific treatment		
Human immunoglobulin	37	90.2
Plasmapheresis	0	0
No specific treatment	4	9.8
Median number of days between onset of strength loss and specific treatment	6	
Use of mechanical ventilation	6	14.6
Admission to the intensive care unit	7	17.1
Lethality	1	2.4

*Huges et al. 1978. **Other: Trigeminal, vestibulocochlear, accessory. ***Other: One pregnant patient, one puerperium patient, one confirmed dengue by NS1 before admission, two patients with rash, and two patients with myalgia and arthralgia. **AMAN:** acute motor axonal neuropathy; **AMSAN:** acute sensorimotor axonal neuropathy; **AIDP:** acute demyelinating inflammatory polyneuropathy; **SMF:** Miller-Fisher syndrome.

Thirty-four patients (83%) had events prior to the onset of weakness that may have triggered GBS: 16 patients (39%) had an infection of the upper respiratory tract; 13 patients (32%) had gastroenteritis; patients (10%) had a recent vaccination (2 received a tetanus vaccine, 1 received an influenza vaccine, and 1 received a vaccine against hepatitis B); 7 patients (17%) had other events, including 1 pregnant patient, 1 patient in the puerperium, and 1 patient with dengue confirmed by serology before admission to the study, 2 patients had a rash, and 2 patients had myalgia and arthralgia. The median period between the event with triggering potential and the onset of weakness was 7 days.

The symptoms and exposure factors that suggested arbovirus infection were fever (32%), diarrhea (20%), skin rash or rash (7%), arthralgia (7%), and mosquito bite (5%). The "seasonal tropical climate" division of cases was 70% during the rainy season and 41% during the dry season.

The pain was reported by 46% of the patients during admission. Meningism was not observed in this study. The most affected sites were the legs (27%), dorsal region (29%), arms (22%), neck (7%), ventral region (5%), face (2%), and other sites (5%)**.** Cranial nerve involvement was observed in 54% of the cases, of which 34% involved facial nerves, 29% involved bulbar nerves, 7% involved oculomotor nerves, and 7% involved other nerves.

Fifty-one percent of the patients had preserved cervical strength upon admission. Lower limb weakness was more severe than upper limb weakness. The paresis of the upper and lower limbs had a predominance of Grade 4 strength according to the Medical Research Council strength scale. Most patients had areflexia, and 54% had sensory deficits. The legs were most affected by sensory loss (46%), followed by the arms (22%) and trunk, vertex, and face (5%). Deficits in pain, vibration, and tactile sensitivity were equally prevalent in 27% of the patients. Ataxia was observed in only 15% of the patients tested (71%).

Autonomic dysfunction was present in 42% of the patients, with changes in blood pressure (20%), bladder dysfunction (17%), gastroenteric dysfunction (7%), and cardiac dysfunction (5%).

Eighty-three percent had the classic form without variants, 2% had FCB, 2% had SMF, 7% had SGB-SMF overlap, and 5% had SMF-FCB overlap. There were no cases of paraparetic GBS (Wakerley classification)^9^.

 Thirty-nine patients underwent electromyography (EMG) during admission. AIDP was found in 53.8% of cases, AMAN in 23%, acute AMSAN in 20.5%, and an undetermined form in 2.5%.

**FIGURE 1:**
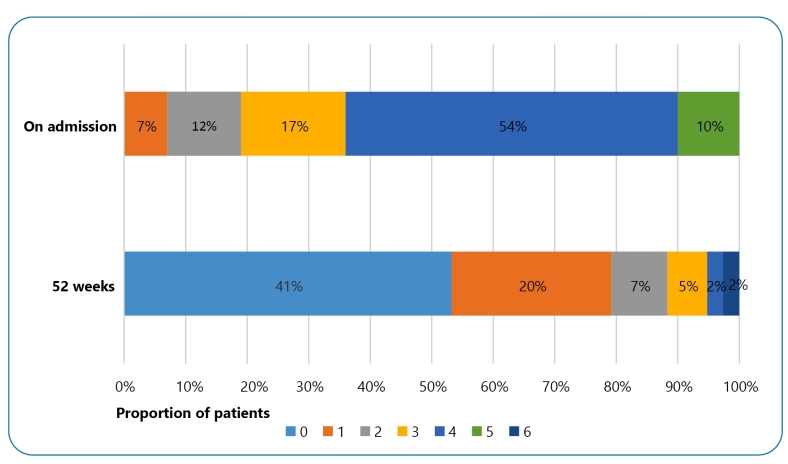
Degree of disability observed upon admission and compared with the last follow-up evaluation (52 weeks) in patients with GBS treated at the IHBDF from May 2017 to April 2019.

Forty-one percent of the patients exhibited Grade 0 disability in their last evaluation (52 weeks) and showed complete improvement of symptoms after at least 1-year of follow-up (Figure 1). In addition, a significant decrease in disability was observed.

Ninety percent received human immunoglobulin, and 10% received no specific treatment. None of the patients underwent plasmapheresis. The median time between the onset of symptoms and the beginning of treatment was 6 days. There was a fluctuation of symptoms (relapse) in 6% of the patients, and a new human immunoglobulin cycle was performed.

ICU admission occurred in 17% of the patients, and 15% required mechanical ventilation. The median length of ICU stay was 8 days. There was one death due to probable brainstem involvement by BE with dysautonomia^18^.

**FIGURE 2:**
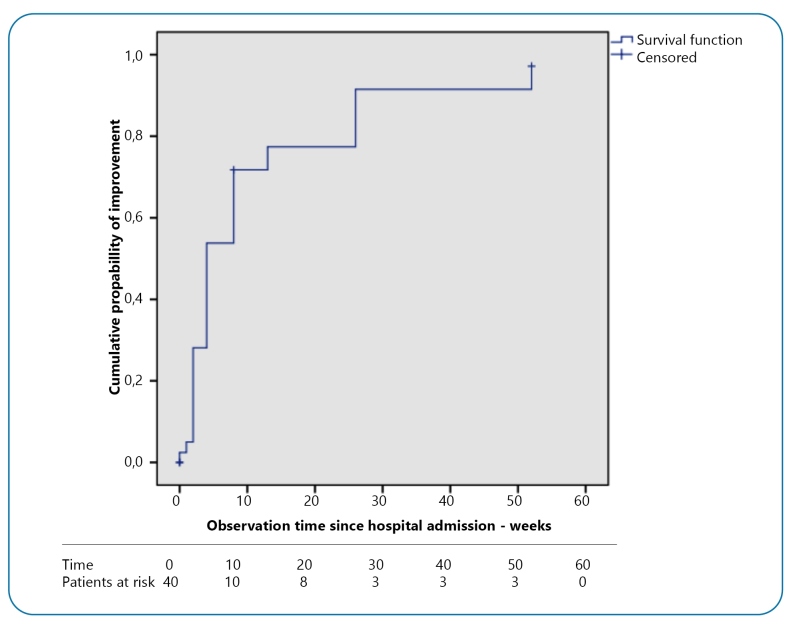
Cumulative probability of improvement (Kaplan-Meier method) until the outcome of improvement in patients with GBS treated at the IHBDF from May 2017 to April 2019.

Figure 2 shows the cumulative probability of patient survival to the improvement outcome, defined as a 1-point reduction in the degree of disability over the observation period. Most patients showed improvement until the 10th week of observation, and the median time until the onset of improvement was 4 weeks (95% CI 1.1 to 6.2).

**FIGURE 3:**
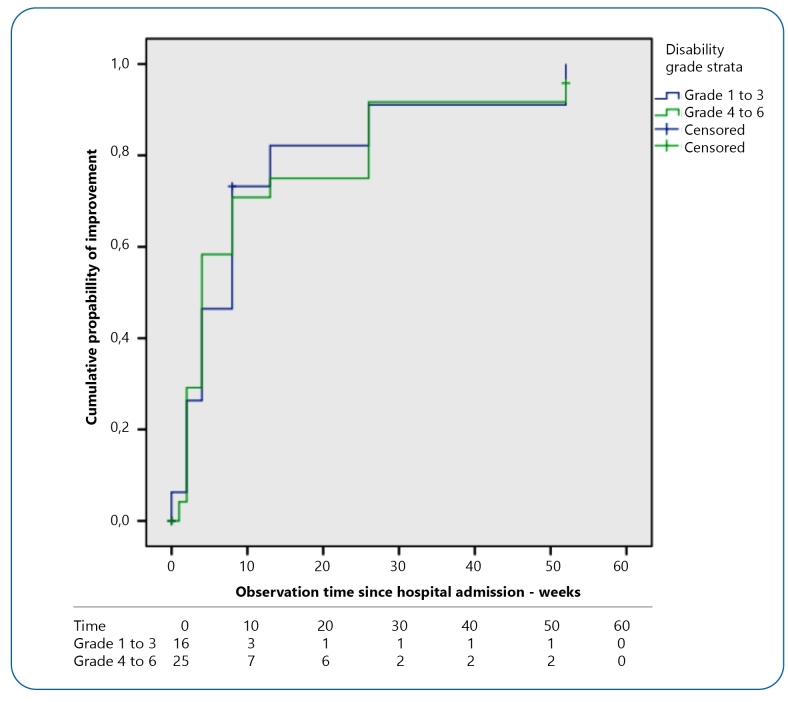
Cumulative probability of improvement (Kaplan-Meier), according to the degree of disability, until the outcome of disability improvement in patients with GBS treated at the IHBDF in the period from May 2017 to April 2019.

Figure 3 shows the patients were divided into two strata, 1 to 3 and 4 to 6, which had similar times for the improvement outcome, defined as a reduction of 1 point in the degree of disability, throughout the observation period regardless of the initial degree of disability (log-rank = 0.013; *P* = 0.908).

None of the patients had positive RT-PCR results for ZIKV, DENV, or CHIKV after admission or positive serology for ZIKV and CHIKV. However, four patients had positive immunoglobulin M (IgM) serological results for dengue. Table 2 shows the characteristics of patients with GBS associated with DENV infection.

**TABLE 2:** Characteristics of four patients with GBS associated with dengue virus infection treated at a tertiary referral center from May 2017 to April 2019 in Brasília, Federal District, Brazil.

Patient	**Sex, Age**	**Diagnosis of the infectious event** ^1^	**Previous events**	**Symptoms of preceding arbovirus infection**	**Neurological characteristics at admission** ^2^	Study of nerve conduction	**Cerebro spinal flui**d	**Treatment**	**Evolution** **at 52 weeks of follow-up**
1	F 60 years	Dengue IgM + upon admission	Common cold Gastroenteritis	Skin *rash* Diarrhea	Disability scale 3 Presence of sensory deficit, pain, cranial nerve involvement, ataxia; Absence of autonomic dysfunction Classical GBS	AIDP^3^	Protein 0,12 g/L Cells: 0/µL	Human immunoglobulin IV	Absence of complications Disability scale 1
2	M 12 years	Dengue IgM + upon admission	None	None	Disability scale 2 Presence of pain Absence of sensory deficit, cranial nerve involvement, ataxia, autonomic dysfunction Classical GBS	AIDP	Not performed	Not performed	Absence of complications Disability scale 0
3	M 33 years	Dengue IgM + upon admission	Dengue	Mosquito bite Fever Skin ras*h* Arthralgia	Disability scale 4 Presence of autonomic dysfunction (blood pressure and bladder dysfunction), cranial nerve involvement, ataxia; Absence of pain, sensory deficits Classical GBS	AIDP	Protein 0.43 g/L Cells: 3/µL	Human immunoglobulin IV	Absence of complications Disability scale 0
4	M 42 years	Dengue IgM + upon admission	None	None	Disability scale 5 Presence of pain, autonomic dysfunction (blood pressure); Absence of cranial nerve involvement Impossible to examine: ataxia, sensory deficits Classical GBS	Not performed	Protein 0.74 g/L Cells: 1/µL	Human immunoglobulin IV	Complications: intensive care unit admission and mechanical ventilation. Disability scale 3

^1^ IgM serology for Dengue virus using Mac-ELISA. Huges et al. (1978) disability scale. **AIDP:** acute demyelinating inflammatory polyradiculopathy; **ICU:** intensive care unit; **M:** male; **F:** female.

## DISCUSSION

The present clinical cohort described the clinical, laboratory, and electromyographic profiles of patients with GBS in Brasília, Brazil, monitored over 2 years. The current study characterized the clinical course, subtypes, and outcomes of GBS with a 1-year follow-up period. We successfully identified four patients with recent exposure to DENV infection as a potentially relevant triggering event.

Observations over 52 weeks showed that most patients showed improvement during the first 10 weeks of evolution.

The present study found that the mean age of patients with GBS was 40 years, slightly below the mean age of 51 years in other studies in South America, Asia, and the IGOS Consortium. The male-to-female ratio was 1.5:1, consistent with the literature ^25^ ^-^ ^31^. Previous events were characterized in 83% of cases, similar to other studies^25^ ^,^ ^32^.

Pain frequency was consistent with that reported in a study in Denmark (55%), but much higher than in the study in Thailand^32^ ^,^ ^28^.

Cranial nerve involvement was observed in 54% of cases, similar to the percentage reported in published reviews. However, ophthalmoparesis was present in only 7% of the patients, well below the reported value of 20%^33^.

Some degree of hyporeflexia/areflexia was found in 100% of the patients. The prevalence was 98% and 90% in French and Thai studies, respectively^10^ ^,^ ^28^. A previous review found sensory deficits in 54% of the patients, higher than expected^31^ but similar to other studies (53.3%, 78%)^28^ ^,^ ^32^.

In addition, in 42% of the patients, autonomic dysfunction was higher than reported in the literature (10%, 17%)^28^ ^,^ ^32^.

The classic form was the most common (83%), and a higher value was observed compared to other studies (70% and 69%, respectively)^34^ ^,^ ^10^. Only 2% of the patients had FCB and pure SMF forms. These rates vary greatly across countries. SMF frequency is 8.7% in Canada, 8% in France, 6.7% in Thailand, 17% in Japan, and 10% in Denmark^10^ ^,^ ^28^ ^,^ ^32^ ^,^ ^34^. The FCB form has been reported in the literature in 2%, 6.7%, and 1.9% of cases^10^ ^,^ ^28^ ^,^ ^35^. SGB-SMF overlap was observed in 7% of our cases, and SMF-FCB overlap was observed in 2%. There have been few reports of these forms in other studies. A previous Japanese study reported a rate lower than 1%^34^.

The most common form in nerve conduction studies was AIDP, consistent with the literature (58% to 66.7%)^25^ ^,^ ^27^ ^,^ ^28^ ^,^ ^35^ ^,^ ^36^. The AMAN and AMSAN rates were 17% and 15%, respectively, consistent with a study in Chile that reported 26.7% axonal forms^27^.

Upon admission, the most common degree of disability was Grade 4 (56%). Most cases were classical GBS and AIDP subtypes, similar to Europe and America^25^. Of the demyelinating subtypes, 61% had a degree of disability greater than or equal 4. The most frequent degree of disability was 4, and 51.2% described previous infectious conditions^27^.

Forty-nine percent of cases in the outcome evaluation had a degree of disability of 0 or 1, similar to the results of Thai and Canadian studies^28^ ^,^ ^35^. The last evaluations were performed via videoconference in some cases because of the COVID-19 pandemic.

All 37 treated patients received human immunoglobulins. Four patients did not undergo any specific treatment because they were treated in the acute phase at another hospital. The need for mechanical ventilation (15%) was well below the needs reported in a review study (30%) and GBS during the ZIKV outbreaks in Salvador, Brazil (22%) but consistent with a study in Thailand (13.3%)^17^ ^,^ ^31^ ^,^ ^38^ ^,^ ^40^.

Thirty-four patients (83%) had previous GBS events, which may be a triggering factor for immunological changes responsible for the syndrome’s pathophysiology. Twenty-nine patients (61%) had events of probable infectious etiology (upper respiratory tract infection and gastroenteritis/diarrhea), supporting the importance of infectious diseases as triggers for the process.

Gastrointestinal symptoms have been reported in the context of other ZIKV outbreaks, and these symptoms may be underrecognized clinical features of ZIKV disease^18^.

Symptoms and factors of exposure and history of the presentation of symptoms suggestive of infection by the three arboviruses studied were reported by 71% of the patients. However, studying the association between ZIKV, DENV, and CHIKV infections and GBS is challenging because these viruses have short viremia periods that reduce the opportunity for detection, and the available serological tests do not have adequate accuracy^39^ ^,^ ^40^.

However, not all patients likely described the preceding symptoms, and these patients did not provide laboratory evidence of ZIKV infection.

Four patients had positive serology results, which confirmed a recent DENV infection. There was no detectable relationship between specific laboratory tests for ZIKV and CHIKV. Perhaps the small and decreasing number of cases of ZIKV infection in Brasília, Brazil, explains this finding because we expected to observe some cases associated with this infection in Brazil and America. Brazil has the highest incidence of ZIKV infection worldwide^37^, and a higher incidence of GBS is expected in these patients^40^, as reported by Stycznski^18^ and Silva^17^. However, we did not observe a large number of cases of ZIKV infection or GBS associated with this virus in Brazil during the study period.

The incidence of infections by Zika and Chikungunya in Brasília and Brazil was 2.7 cases/100,000 inhabitants and 5.1 cases/100,000 inhabitants in 2017, 1.6 cases/100,000 inhabitants and 2.6 cases/100,000 inhabitants in 2018, and 2 cases/100,000 inhabitants and 1.2 cases/100,000 inhabitants in 2019, respectively (Official Government Epidemiological Report SVS/SES-DF).

The study's main limitation was the small sample size, which did not allow for a more detailed exploration of factors associated with prognosis. However, the cohort primarily consisted of patients with GBS treated in the public network of the Federal District over 2 years. The lack of access to patients treated in the private network is also a limitation because factors such as socioeconomic stratum may cause selection bias and affect the validity of the results. In addition, patients were not tested for *Campylobacter* infection, and this etiology is a triggering factor that cannot be excluded as a relevant factor for patients with recent exposure to DENV.

The data presented are useful in building local knowledge about GBS and providing a warning about the potential role of arboviruses in the pathogenesis of the disease.
